# Golgi phosphoprotein 3 (GOLPH3) promotes hepatocellular carcinoma progression by activating mTOR signaling pathway

**DOI:** 10.1186/s12885-018-4458-7

**Published:** 2018-06-18

**Authors:** Hongying Liu, Xieqi Wang, Bing Feng, Lipeng Tang, Weiping Li, Xirun Zheng, Ying Liu, Yan Peng, Guangjuan Zheng, Qinglian He

**Affiliations:** 1 Department of Pathology, The Second Clinical College of Guangzhou University of Chinese Medicine,Guangdong Provincial Hospital of Chinese Medicine, 111 Dade Road, Guangzhou, 510120 Guangdong China; 2 Department of Pharmacology of Traditional Chinese Medicine, The Second Clinical College of Guangzhou University of Chinese Medicine,Guangdong Provincial Hospital of Chinese Medicine, 111 Dade Road, Guangzhou, 510120 Guangdong China; 3 Guangzhou University of Chinese Medicine, 232 Waihuan East Road, Guangzhou, 510006 China

**Keywords:** GOLPH3, Hepatocellular carcinoma, mTOR signaling pathway

## Abstract

**Background:**

Hepatocellular carcinoma (HCC) is the sixth most common cancer and the second leading cause of cancer-related deaths worldwide. Despite new technologies in diagnosis and treatment, the incidence and mortality of HCC continue rising. And its pathogenesis is still unclear. As a highly conserved protein of the Golgi apparatus, Golgi phosphoprotein 3 (GOLPH3) has been shown to be involved in tumorigenesis of HCC. This study aimed to explore the exact oncogenic mechanism of GOLPH3 and provide a novel diagnose biomarker and therapeutic strategy for patients with HCC.

**Methods:**

Firstly, the expression of GOLPH3 was detected in the HCC tissue specimens and HCC cell lines. Secondly, RNA interference was used for GOLPH3 gene inhibition. Thirdly, cell proliferation was analyzed by MTT; cell apoptosis was analyzed by Annexin-V/PI staining, Hoechst 33,342 staining and caspase 3/7 activity assay. Fourthly, xenograft tumor model was used to study the function of GOLPH3 in tumor growth in vivo. Finally, western blotting and immunohistochemistry were used to investigate the role of GOLHP3 in the mTOR signaling pathway.

**Results:**

Data showed that the mRNA and protein expression of GOLPH3 were up-regulated in HCC tumor tissue and cell lines compared with those of control (*P* < 0.05). Correlation analyses showed that GOLPH3 expression was positively correlated with serum alpha-fetoprotein level (AFP, *P* = 0.006). Knockdown GOLPH3 expression inhibited proliferation and promoted apoptosis in HCC cell lines. What’s more, knockdown GOLPH3 expression led to tumor growth restriction in xenograft tumor model. The expression of phosphorylated mTOR, AKT and S6 K1 were significantly higher in HCC tumor tissue and cell lines compared with those in normal liver tissues (*p* < 0.05). While the phosphorylated mTOR, AKT and S6 K1 were much lower when diminished GOLPH3 expression in HCC cell lines both in vitro and in vivo.

**Conclusion:**

The current study suggests that GOLPH3 contributes to the tumorigenesis of HCC by activating mTOR signaling pathway. GOLPH3 is a promising diagnose biomarker and therapeutic target for HCC. Our study may provide a scientific basis for developing effective approaches to treat the HCC patients with GOLPH3 overexpression.

**Electronic supplementary material:**

The online version of this article (10.1186/s12885-018-4458-7) contains supplementary material, which is available to authorized users.

## Background

Hepatocellular carcinoma (HCC) is one of the most common solid tumors with second cancer-related lethal rate worldwide [1]. Compared with the most solid cancers, the incidence and mortality of HCC have increased over the past decades in many parts of the world. Especially in China, HCC accounts for one-fifth of the incidence of malignant tumors [2]. In clinic, HCC can be treated with surgical resection, liver transplantation, interventional therapy, liver directed therapy and systemic therapy. Among these options, only surgical resection and liver transplantation are considered as potentially curative approaches. However, only 15% patients are eligible with effective treatment while a majority is present with advanced disease [3]. Therefore, developing more efficient therapies that based on the precise pathogenesis of HCC is critical for the clinical management of HCC. With rapid advances in the molecular biology of HCC, several prognostic biomarkers are identified, including alpha fetoprotein (AFP) [4, 5], glypican-3 [6], des-γ-carboxyprothrombin (DCP) [7], cytokeratin 19 [8] and so on. Even though these biomarkers are used widely in some countries, there are still shortages for clinical diagnosis and prognosis predication. It is reported that elevation in AFP level is not evident in around 80% of small HCC [9]. In HCC patients, the specificity of serum DCP-based diagnosis was 81–98%, but the sensitivity was only 48–62% [10]. And recent studies have showed that cell-free DNA (cfDNA) is associated with oncogenesis and cancer progression, which can be used as a biomarker [11]. However, clinical significance of cfDNA needs to be futher confirmed. So, these limitations highlight the necessity and urgency to find additional biomarkers that can be used individually or combined with other markers for HCC diagnosis.

Known as GPP34/GMx33/MIDAS in animal, Golgi phosphoprotein 3 (GOLPH3) is a highly conserved phosphorylated protein expressed on the Golgi apparatus [12]. It is encoded by a gene residing on human chromosome 5p13, which is frequently amplified in multiple solid tumor types [13]. Recent studies have demonstrated that GOLPH3 is an oncogene involved in the development and progression of several tumor types including lung cancer [14, 15], breast cancer [16], melanoma [13], colon cancer [17], bladder cancer [18], gastric cancer [19, 20], prostate cancer [21], oral tongue cancer [22], rhabdomyosarcoma [23], glioma [24] and so on. Especially several studies have demonstrated that high expression of GOLPH3 is associated with poor survival in patients with HCC [25, 26]. However, the exact pathological mechanism of GOLPH3 in the tumorigenesis of HCC is still ambiguous. A better understanding of the activity and mechanism of GOLPH3 will contribute to explore cancer pathogenesis, and provide novel targets and therapeutic strategies for patients with HCC. It has been elucidated that GOLPH3 can regulate cell size, enhance growth-factor-induced mTOR signaling in many human cancer cell lines like A549 and CRL-5889, and alter the response to rapamycin which is a mTOR inhibitor in vivo [13]. However, whether GOLPH3 can promote HCC progression by activating mTOR signaling is still unknown. The objectives of this study were to investigate the relationship between GOLPH3 and mTOR signaling pathway in HCC.

## Methods

### Patients and tissue samples

The HCC tissue specimens, including HCC tissues and adjacent normal liver tissues, were obtained from Guangdong Provincial Hospital of Chinese Medicine (GPHCM) between 2011 and 2016. None of the patients who participated in this study received any pre-operative treatments, including TACE or radiofrequency ablation. Their clinicopathological characteristics were collected. Histological type and grade were established according to the World Health Organization (WHO) standards and further independently evaluated by two experienced pathologists. Immunohistochemistry (IHC) and quantitative real-time PCR (qPCR) were used to analyze the expression of GOLPH3 in both tumor tissues and adjacent normal liver tissues (not less than 2 cm away from the tumor tissues). This study was approved by the Ethic Committee of GPHCM.

### Cells and cell cultures

Hepatocellular carcinoma cell lines, including Bel-7402 (catalogue number TCHu 10), QGY-7703 (catalogue number TCHu 43), MHCC97L (catalogue number CC0109), MHCC97H (catalogue number SCSP 528) were cultured in RPMI 1640 (Gibco BRL, Rockville, MD) supplemented with 10% fetal bovine serum (Gibco BRL, Rockville, MD), penicillin (100 units/ml) and streptomycin (100 units/ml), and maintained in a 5% CO_2_-humidified incubator at 37 °C. All cell lines were purchased from the Cell Bank of the Chinese Academy of Sciences (Shanghai, China).

### Immunohistochemistry (IHC) analysis

IHC analysis on 4 μm paraffin-embedded specimens sections was performed by using Ventana Benchmark autostainer (Roche Diagnostics, Indianapolis, IN, USA), according to the best protocol for each antibody tested in our laboratory. The primary antibody for mTOR (catalogue number 2983), phospho-mTOR (Ser2448) (catalogue number 5536), AKT (catalogue number 4691), phospho-AKT (Ser473) (catalogue number 4060), S6 K1 (catalogue number 2708), phospho-S6 K1 (Thr389) (catalogue number 9234) were purchased from Cell Signaling Technology (CST) (Boston, MA, USA), GOLPH3 (catalogue number ab98023) was purchased from Abcam (Cambridge, MA, USA). Slides were viewed and photographed under a light microscope, and analyzed by using Image-Pro Plus software (version 6.2) program (Media Cybernetics). The intensity of staining was graded as follows: 0 (no staining), 1 (weak staining, light yellow), 2 (moderate staining, yellow brown) and 3 (strong staining, brown). The proportion of positive tumor cells were scored according to the following standards: 1 (< 10%), 2 (10–35%), 3 (35–70%) and 4 (> 70%). The staining index was determined by multiplying the intensity score by the proportion score. A staining index score of ≥6 was designated as tumors with high GOLPH3 expression, whereas a score of ≤4 was designated as low GOLPH3 expression.

### Quantitative real-time PCR analysis (qPCR)

Total RNA from formalin-fixed paraffin-embedded tissues was extracted by using nucleic acid extraction kit (AmoyDx, China) according to the manufacturer’s protocol. And total RNA was extracted from cultured cells by using the RNAiso plus reagent (TaKaRa, Japan). Total RNA was reverse transcribed by using Primescript™ RT reagent kit gDNA Eraser (TaKaRa, Japan). qPCR was performed by using SYBR Premis Ex Tag™II(TaKaRa, Japan) on the 7500 qPCR System (Applied Biosystems Inc., Foster City, CA, USA).The qRCR reactions were pre-incubated for 10 min at 95 °C, followed by 40 cycles of denaturation for 30 s at 95 °C and annealing for 1 min at 60 °C. The primers for amplifying GOLPH3 are as follows: 5’-GGGCGACTCCAAGGAAAC-3′ (forward) and 5’-CAGCCACGTAATCCAGATGAT-3′ (reverse), and primers to amplify GAPDH contains: 5’-AGCCACATCGCTCAGACACC-3′ (forward) and 5’-CGCCCAATACGACCAAATCC-3′ (reverse). Reactions containing either no reverse transcriptase or no template were used as negative controls, and all reactions were performed in triplicate. The GAPDH was used as the normalization control, and the relative expression level was calculated using the 2 − ^ΔΔCT^ equation.

### Western blotting analysis

Cells were washed twice with PBS and lysed by using RIPA lysis buffer (CWBIO, China) containing protease inhibitor cocktail (Thermo, Rockford, IL, USA) and phosphatase inhibitor (Calbiochem, Billerica, MA, USA). The extracts were then subjected to centrifugation at 15,000×g for 30 min at 4 °C. And protein concentration was determined by using BCA protein assay kit (Thermo, Rockford, IL, USA). Total proteins (40 μg) from each sample with loading buffer were heated at 100 °C. Samples were separated by using SDS-PAGE and electro-transferred to polyvinylidene fluoride membranes (Millipore, Billerica, MA, USA). Membranes were blocked with TBS-T containing 5% skimmed milk at room temperature for 1 h. Membranes were then incubated with primary antibodies in TBS-T overnight at 4 °C on shaker. Washed with TBS-T, the membranes were incubated secondary antibody for 1 h at room temperature following day. The expression of proteins were detected with the Molecular Imager ChemiDoc XRS and Image Lab 5.2.1 software (Bio-Rad Laboratories, Hercules, USA) following various exposure times. And Image J software was used to quantify the density of the bands. For western blotting, primary polyclonal antibodies against GOLPH3 (1:2000 dilution), mTOR (1:2000 dilution), p-mTOR (1:2000 dilution), AKT (1:2000 dilution), p-AKT (1:2000 dilution), S6 K1 (1:2000 dilution), p-S6 K1 (1:2000 dilution), Raptor (1:1000 dilution, Proteintech, catalogue number 20984–1-AP), Rictor (1:1000 dilution, Santa Cruz, catalogue number sc-271081), DNA-PKcs (1:1000 dilution, Santa Cruz, catalogue number sc-390849), Ku70 (1:1000 dilution, Santa Cruz, catalogue number sc-17789) and Ku86 (1:1000 dilution, Santa Cruz, catalogue number sc-5280) were used. GAPDH antibody (1:5000 dilution, CST, catalogue number 5174) and β-actin (1:5000 dilution, CST, catalogue number 4970) served as the internal control.

### RNA interference

For transient knockdown GOLPH3, MHCC97L and MHCC97H cells were grown in 6-well plates and transfected with GOLPH3 siRNA (si-GOLPH3) (Guangzhou Ribo Biotechnology Co., Ltd.) or a control siRNA (si-Ctrl) (Guangzhou Ribo Biotechnology Co., Ltd.) by using Lipofectamine RNAiMAX reagent (Invitrogen, USA) according to the manufacturer’s instructions. To generate stable GOLPH3 knockdown cells, GOLPH3 RNAi lentivirus plasmids were cotransfected into 293 T cells with GV248, Helper 1.0 and Helper 2.0. Viral supernatant fractions were collected at 48 h after transfection and filtered through a 0.45 μm filter followed by infection into MHCC97L and MHCC97H cells together with 5 μg/mL polybrene. After 12 h infection, the medium was replaced with fresh medium containing 2 μg/mL puromycin and cells were incubated for another 6 days. Western blotting and qPCR were used to verify down-regulated GOLPH3.

### 3-(4,5-dimethylthiazol-2-yl)-2,5-diphenyltetrazolium bromide (MTT) assays

Cells (5 × 10^3^ cells/well) were plated into 96-well plates and each well was incubated with 20 μl 5 mg/ml MTT (Sigma-Aldrich) at 37 °C for 4 h. Absorbance was measured at 490 nm by using a microplate reader (Biotek, USA). Cells incubated with culture medium were used as the control group. Each sample was assayed in triplicate.

### Annexin V-FITC/PI staining

Apoptosis assay was performed by Annexin V-FITC/PI apoptosis kit (Multi science, China) according to the manufacturer’s protocol. Transfected HCC cells were washed twice in PBS and resuspended in binding buffer. The cells were then stained with AnnexinV-FITC and propidium iodide (PI) for 5 min in dark and followed by FACS analysis by using FC500 (Beckman Coulter, USA).

### Hoechst 33,342 staining

Cells (1 × 10^4^ cells/well) were plated in 12-well plates and then transfected with si-GOLPH3 or si-Ctrl for 144 h. 10 μl Hoechst 33,342 (5 mg/ml; Beyotime, Jiangsu, P.R.China) was added to plate. After 10 min, cells were washed twice in PBS and observed in a fluorescence microscope (Nikon-TE2000U, Tokyo, Japan).

### Caspase 3/7 activity assay

Caspase-Glo 3/7 assay (Promega, Madison, WI, USA) was used to measure the activation of caspase 3 and caspase 7. Cells (5 × 10^3^ cells/well) were seeded in 96-well white plate. Transfected cells were incubated in the CO_2_ incubator at 37 °C for 144 h. 100 μl Caspase-Glo 3/7 reagent was added and the plate was kept at room temperature for 3 h in dark prior to luminescence detection.

### Xenograft tumor model

Male Balb/c nude mice (aged 4–5 weeks, weight 18–20 g) (Beijing Vital River Laboratory Animal Technology Co., Ltd., Beijing, China) were housed in barrier facilities on a 12 h light/dark cycle. All experimental procedures were approved by the Institutional Animal Care and Use Committee of GPHCM. The Balb/c nude mice were randomly divided into four groups (*n* = 7 for each group), inoculated subcutaneously with 5 × 10^6^ stable LV-Ctrl or LV-GOLPH3-RNAi MHCC97L cells and LV-Ctrl or LV-GOLPH3-RNAi MHCC97H cells. The tumor length (L) and width (W) were measured with calipers every two days, and tumor volumes were calculated by using the eq. (L × W^2^)/2. The animals of MHCC97L groups or MHCC97H groups were sacrificed after five or four weeks respectively and tumor tissues were collected.

### Statistical analysis

Statistical analysis was conducted by using SPSS 20.0 software (International Business Machine, Armonk, NY, USA). Results expressed as mean ± standard deviation (SD) were analyzed by using the Student’s two-tailed t-test. The associations between GOLPH3 protein expression and clinicopathological variables were analyzed by using the chi-square test. The *P* value was based on the two-sided statistical analysis and *P* < 0.05 was considered statistically significant.

## Results

### GOLPH3 is overexpressed in HCC tissue samples and HCC cell lines

**Fig. 1:**
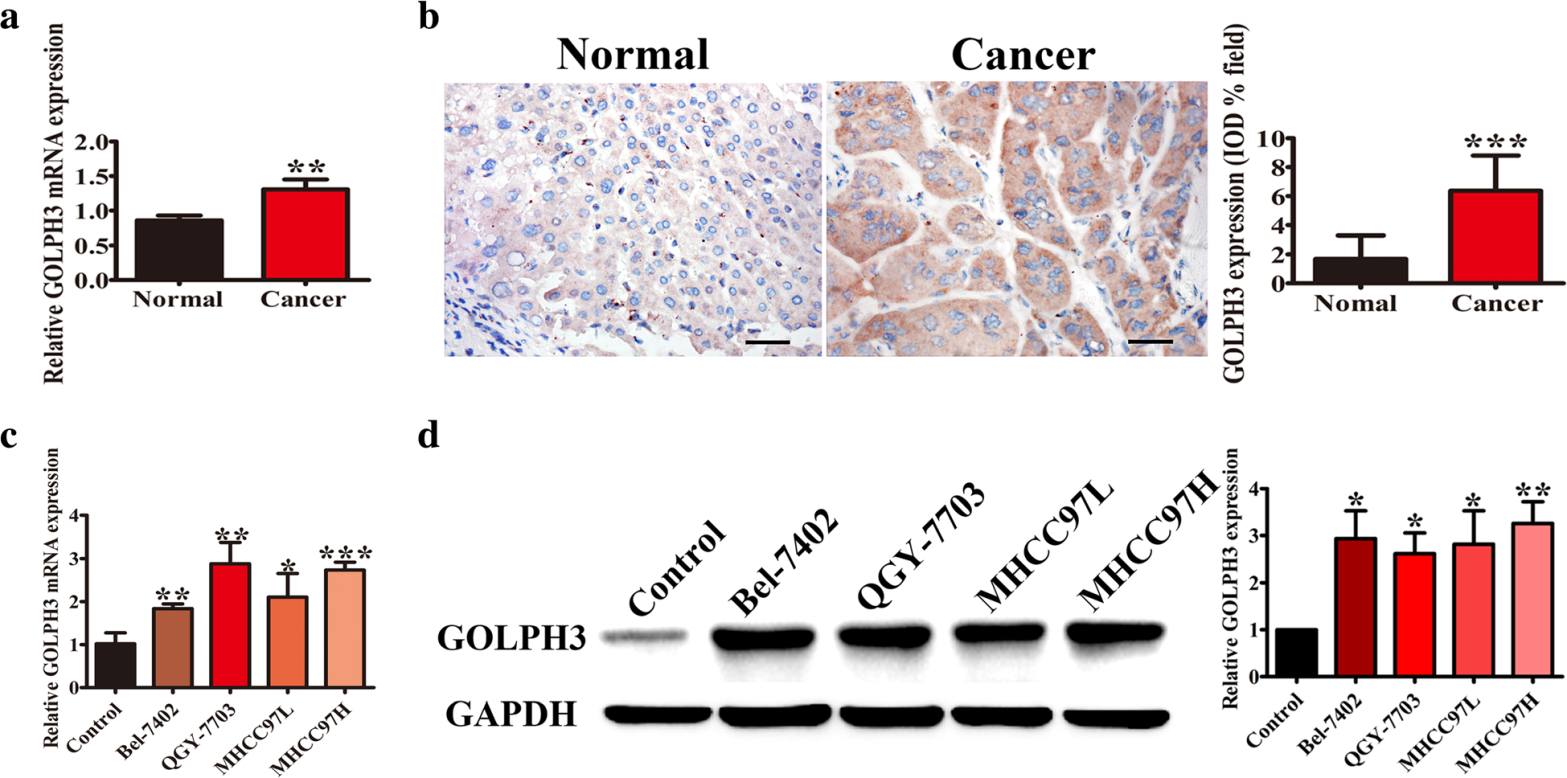
GOLPH3 was overexpressed in tissue samples and HCC cell lines; **a** Expression of GOLPH3 mRNA in 60 pairs of paraffin-embedded HCC and adjacent normal liver samples were examined by qPCR. **b** Expression of GOLPH3 protein in 60 pairs of paraffin-embedded HCC and adjacent normal liver samples were examined by immunohistochemistry. The Magnification of immunohistochemistry images was 400×. **c** Expression of GOLPH3 mRNA of hepatocellular carcinoma cell lines (Bel-7402, QGY-7703, MHCC97L and MHCC97H) was examined by qPCR. **d** Expression of GOLPH3 protein of hepatocellular carcinoma cell lines (Bel-7402, QGY-7703, MHCC97L and MHCC97H) was examined western blotting. Control was a piercing liver tissue which was diagnose as hepatitis without tumor. The expression levels were normalized to GAPDH. Data was presented as the mean ± SD. *P*-values were calculated using Student’s *t*-test, * *P* < 0.05, ** *P* < 0.01, *** *P* < 0.001

To investigate whether the high expression of GOLPH3 is linked to HCC, the expression of GOLPH3 was examined. Firstly, the mRNA and protein expression of GOLPH3 in 60 pairs of paraffin-embedded HCC and adjacent normal liver tissue samples were detected. GOLPH3 mRNA was over-expressed in HCC tumor tissues compared with that in corresponding adjacent normal liver tissues (Fig. 1a). Then, the GOLPH3 protein level of HCC samples were examined by IHC. Results showed that the GOLPH3 protein level of HCC tissues were significantly higher than that of adjacent normal liver tissues (*P* < 0.001) (Fig. 1b). From the statistical data, 42 of 60 (70.0%) samples were classified as HCC with high GOLPH3 protein expression, but only 18 of 60 (30.0%) samples were designated as HCC with low GOLPH3 protein expression (Table 1). This result clearly indicated that most HCC patients had a higher probability of over-expressed GOLPH3 in tumor tissue. The mRNA and protein expression of GOLPH3 were further detected in four HCC cell lines. Result showed that the mRNA and protein of GOLPH3 were much higher in HCC cell lines than those of control (Fig. 1c and Fig. 1d). These results implicated that GOLPH3 might be a critical molecule in HCC development.

### Correlation between GOLPH3 expression and clinicopathological characteristics

The correlation between the expression of GOLPH3 and clinicopathological characteristics of the HCC patients were analyzed by using the chi-square test (Table 1). The expression of GOLPH3 was found to be associated with serum AFP level (*P* = 0.006). It indicated that patients with high GOLPH3 expression had a higher tendency to have higher serum AFP level, which is the main serum marker of HCC. However, no significant correlation was observed between GOLPH3 expression and any other clinicopathological characteristics, such as gender, age, HBsAg, tumor size, vascular invasion and cirrhosis.

**Table 1:** Correlation between GOLPH3 protein expression and clinicopathological characteristics of hepatocellular carcinoma

Clinicopathological characteristics	Number of cases	GOLPH3 expression		*P*-value
		Low	High	
Gender				0.288
Male	39	14	25	
Female	21	4	17	
Age(years)				0.302
≤50	26	6	20	
>50	34	12	22	
Tumor size				0.220
≤5 cm	38	14	24	
>5 cm	22	4	18	
AFP levels				0.006(*)
≤20 ng/ml	21	11	10	
>20 ng/ml	39	7	32	
HBsAg				0.1
negative	27	11	16	
positive	33	7	26	
Vascular invasion				0.186
Absence	36	8	28	
Presence	24	10	14	
Liver cirrhosis				0.071
Absence	31	13	18	
Presence	29	5	24	

*P*-value was calculated by using chi-square test. *P < 0.05 indicates a significant association among the variables

### Knockdown GOLPH3 inhibits the HCC cell proliferation and promotes the HCC cell apoptosis

**Fig. 2:**
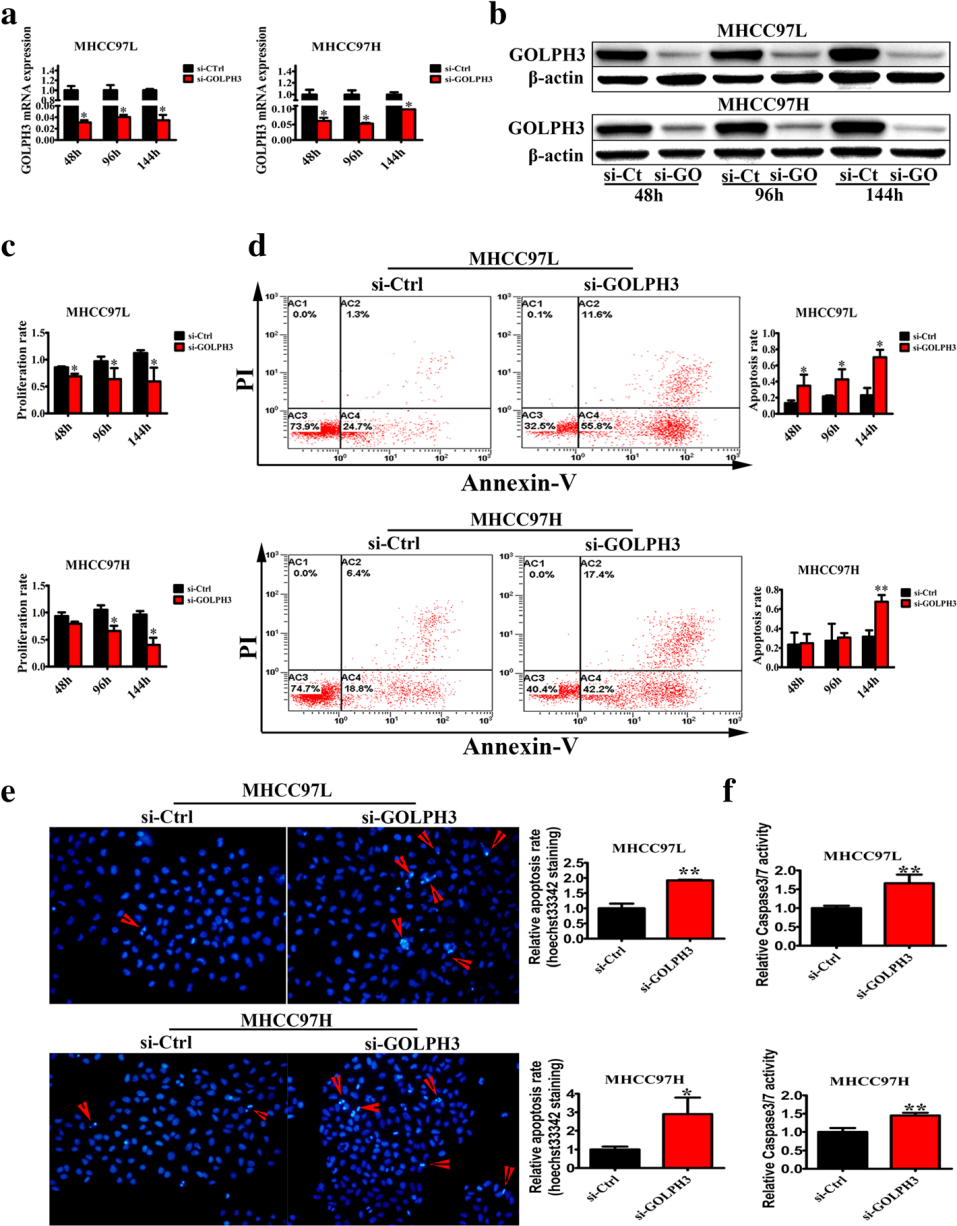
GOLPH3 depletion inhibited the HCC cells proliferation and promoted the HCC cells apoptosis; **a** The relative expression of GOLPH3 mRNA in MHCC97L and MHCC97H cells transfected with si-GOLPH3 for 48 h, 96 h and 144 h. **b** The relative expression of GOLPH3 protein in MHCC97L and MHCC97H cells transfected with si-GOLPH3 for 48 h, 96 h, and 144 h. And si-Ct, si-GO represented si-Ctrl, si-GOLPH3 respectively. **c** MTT assay of HCC cells transfected with si-GOLPH3 for 48 h, 96 h and 144 h. **d** Flow cytometry for Annexin V-fluorescein isothiocyanate (FITC)-propidium iodide (PI) staining of the HCC cells treated with si-GOLPH3 for different time. **e** Hoechst 33,342 staining of HCC cells transfected with si-GOLPH3 after 144 h. The magnification of images was 400×. **f** Caspase3/7 activity assay of the HCC cells after transfected with si-GOLPH3 for 144 h. All experiments were performed in triplicate with at least three independent experiments. Data was presented as the mean ± SD. *P*-values were calculated using Student’s t-test, * *P* < 0.05, ** *P* < 0.01, *** *P* < 0.001, significant difference compared with the si-Ctrl cells in the same time

To examine the function of GOLPH3, we knocked down GOLPH3 gene in MHCC97L and MHCC97H cells by GOLPH3 siRNA. The silence efficiency of GOLPH3 siRNA was verified by qPCR and western blotting. As our data showed, compared with that in the control group, the relative expression of GOLPH3 mRNA and protein was significantly decreased in the GOLPH3 siRNA transfected group at 48 h, 96 h and 144 h (*P* < 0.05) (Fig. 2a and Fig. 2b). MTT assays demonstrated that knockdown of endogenous GOLPH3 expression significantly reduced HCC cells proliferation compared with corresponding control siRNA cells especially at 144 h (Fig. 2c). In addition, Annexin V-fluorescein isothiocyanate (FITC)-propidium iodide (PI) staining indicated that the knockdown of GOLPH3 significantly promoted the apoptosis in MHCC97L and MHCC97H cells compared with control cells at 144 h (Fig. 2d). And this result was further confirmed by Hoechst 33,342 staining (Fig. 2e). Moreover, it was indicated that caspase-3/7, two terminal executioners in apoptosis, were activated in HCC cell lines transfected with GOLPH3 siRNA (Fig. 2f). Taking together, these results demonstrated that diminishment of GOLPH3 expression significantly reduced the tumorigenic properties of HCC cells in vitro.

### GOLPH3 contributes to the progression of HCC in vivo

**Fig. 3:**
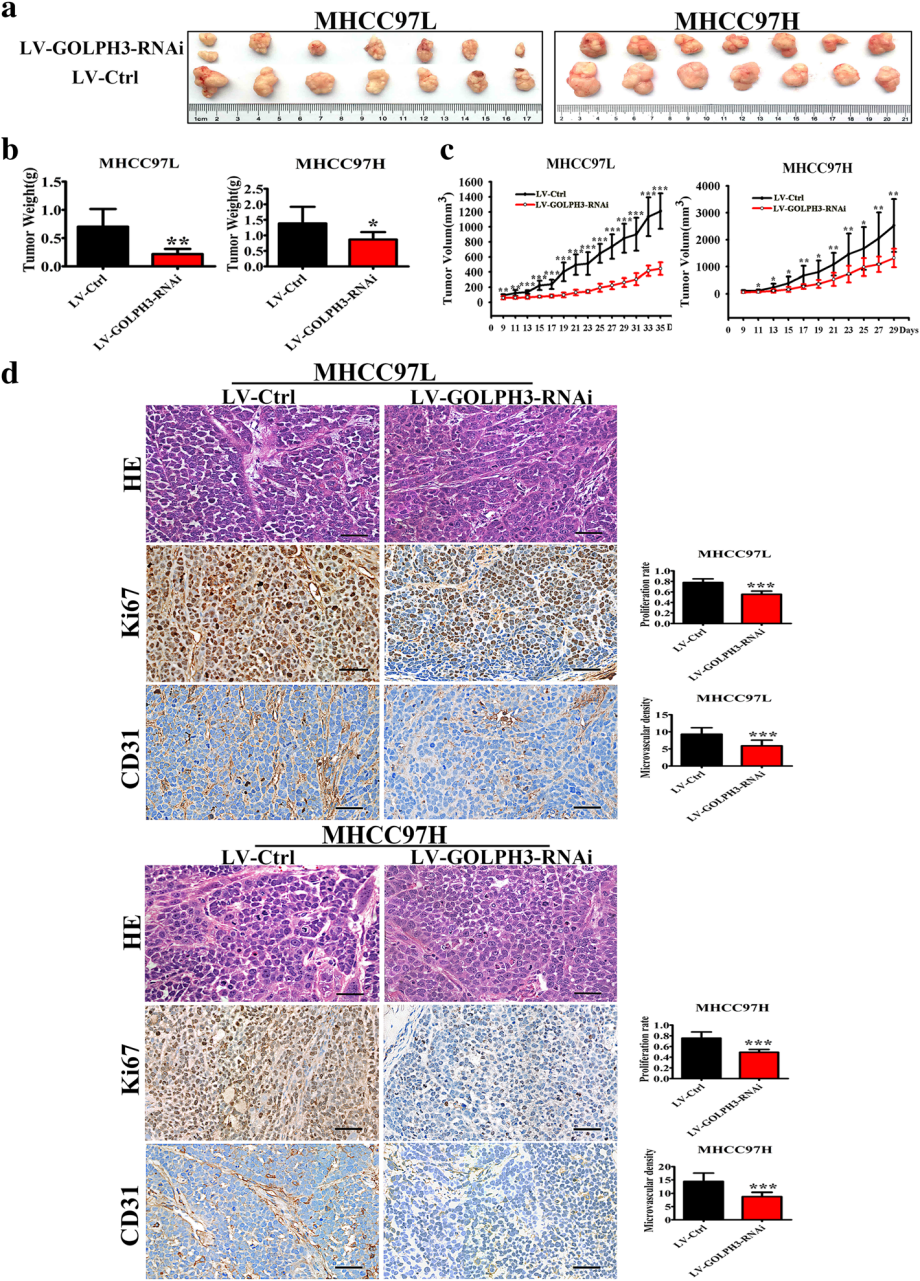
GOLPH3 contributed to the progression of HCC in vivo; **a** Photographs of tumors dissected from mice (*n* = 7) injected with LV-GOLPH3-RNAi or LV-Ctrl HCC cells. **b** Average tumors weight of mice from each group was shown. **c** Volume growth curves for the tumors formed by the HCC cells transfected with LV-GOLPH3-RNAi or LV-Ctrl. **d** HE staining and expression of Ki67 and CD31 in tumors tissue formed by the HCC cells transfected with LV-Ctrl or LV-GOLPH3-RNAi. The magnification of images was 400×. All experiments were performed in triplicate with at least three independent experiments. Data were presented as the mean ± SD. *P*-values were calculated using Student’s t-test, * *P* < 0.05, ** *P* < 0.01, *** *P* < 0.001, significant difference compared with the LV-Ctrl group

The ability of GOLPH3 to promote HCC progression was further examined by using a xenograft tumor model. The stable GOLPH3 knockdown cells were generated. And the results showed that the tumors in the LV-Ctrl group grew faster than those in the LV-GOLPH3-RNAi group (Fig. 3a). The average tumor weights were decreased in the LV-GOLPH3-RNAi group (Fig. 3b). The tumor volumes were decreased in the LV-GOLPH3-RNAi group (Fig. 3c). In addition, IHC staining indicated that both the percentage of Ki67-positive cells and MVD were decreased in GOLPH3 knockdown tumors, which indicated that down-regulation of GOLPH3 inhibited tumor cells proliferation and tumor progression in vivo (Fig. 3d). All these results suggested that knocked down GOLPH3 expression significantly reduced the tumorigenic properties of HCC cells in vivo.

### GOLPH3 activates the mTOR signaling in HCC both in vitro and in vivo

**Fig. 4:**
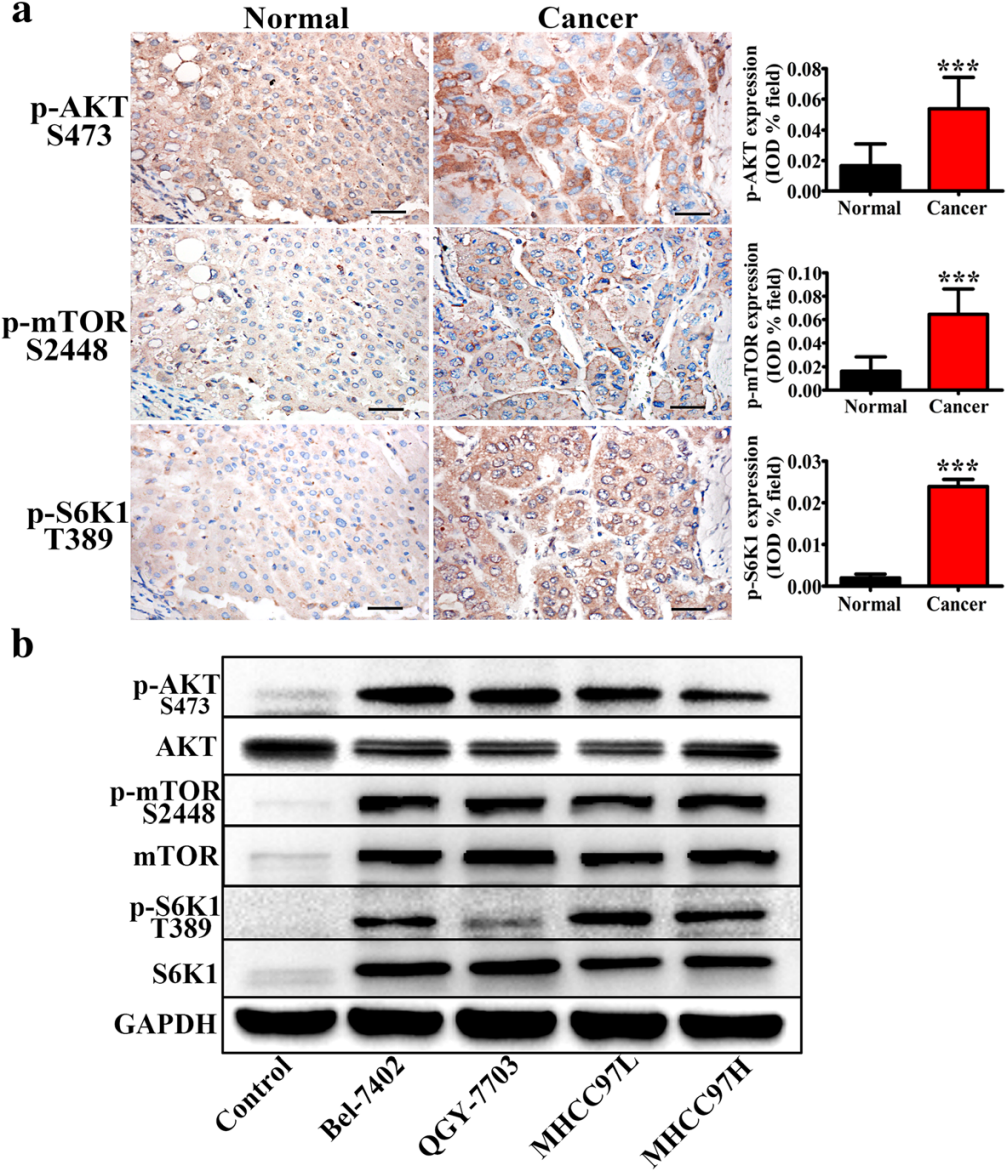
mTOR signaling pathway was activated in HCC; **a** Immunohistochemistry of p-AKT (Ser473), p-mTOR (Ser2448), and p-S6 K1 (Thr389) in HCC clinic samples, the magnification of images was 400×; **b** Western blotting of AKT, p-AKT (Ser473), mTOR, p-mTOR (Ser2448), S6 K1 and p-S6 K1 (Thr389) in hepatocellular carcinoma cell lines (Bel-7402, QGY-7703, MHCC97L and MHCC97H). Control was a piercing liver tissue which was diagnose as hepatitis without tumor. The expression levels were normalized to GAPDH. All experiments were performed in triplicate with at least three independent experiments. Data were presented as the mean ± SD. *P-*values were calculated using Student’s t-test, * *P* < 0.05, ** *P* < 0.01, *** *P* < 0.001

**Fig. 5:**
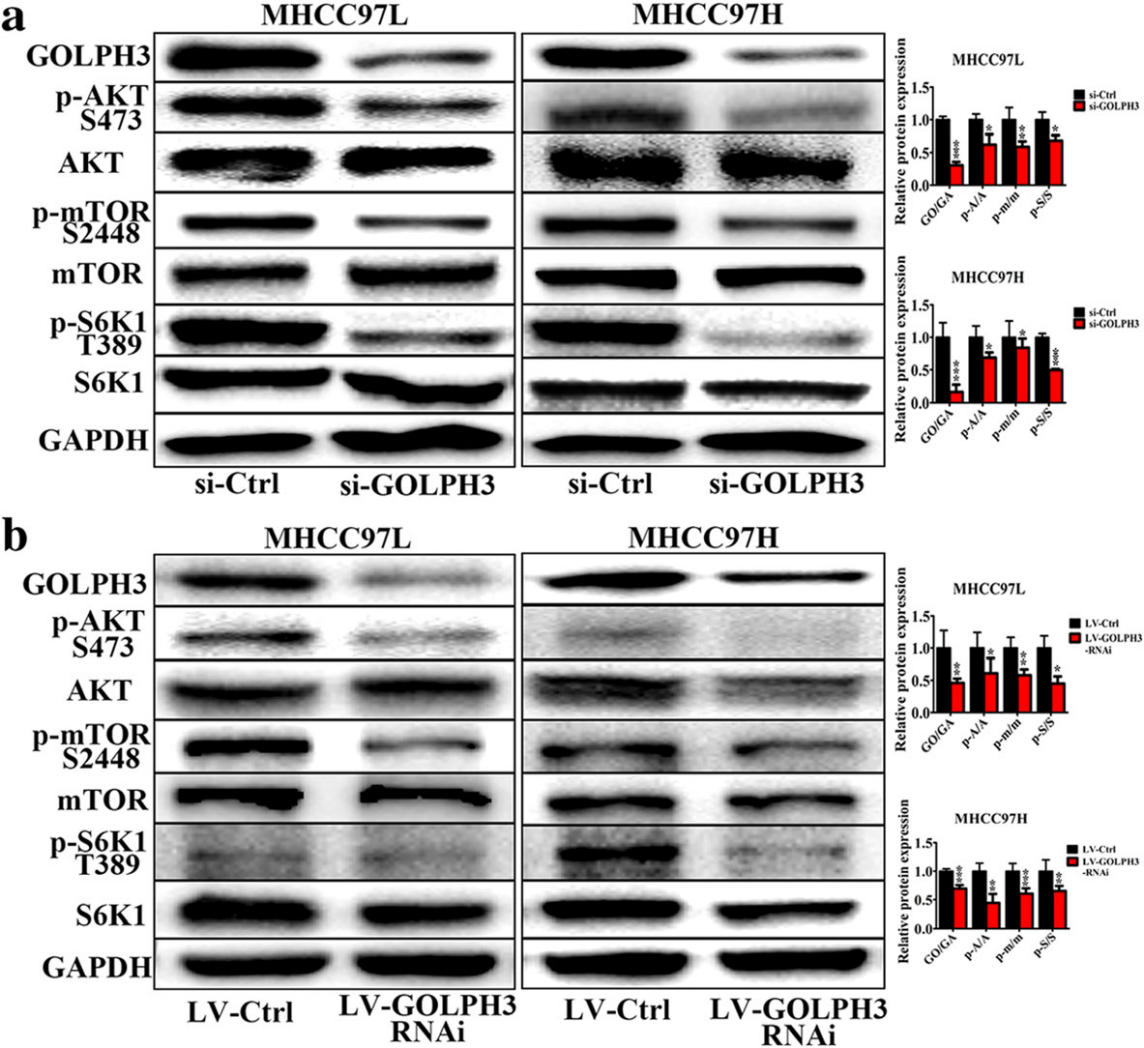
Knockdown of GOLPH3 inactivated mTOR signaling pathway in HCC in vitro and in vivo; **a** Western blotting of AKT, p-AKT (Ser473), mTOR, p-mTOR (Ser2448), S6 K1 and p-S6 K1 (Thr389) in MHCC97L and MHCC97H cells transfected with si-Ctrl and si-GOLPH3. **b** Western blotting of AKT, p-AKT (Ser473), mTOR, p-mTOR (Ser2448), S6 K1 and p-S6 K1 (Thr389) in Xenograft tumor model injected with LV-Ctrl or LV-GOLPH3-RNAi HCC cells. The expression levels were normalized to GAPDH. In Column diagram, GO, GA, A, p-A, m and p-m means GOLPH3, GAPDH, AKT, p-AKT, mTOR and p-mTOR respectively. All experiments were performed in triplicate with at least three independent experiments. Data were presented as the mean ± SD. *P*-values were calculated using Student’s t-test, * *P* < 0.05, ** *P* < 0.01, *** *P* < 0.001

It is reported that GOLPH3 can promote tumor growth via activating mTOR signaling pathway [13]. To test whether GOLPH3 could also promote the progression of HCC by activating mTOR signaling pathway, we examined the expression of key proteins involved in mTOR singling pathway including mTOR, AKT and S6 K1. Firstly, the expression of phosphorylated-mTOR, AKT and S6 K1 in HCC tissue specimens and cell lines were investigated by IHC and western blotting respectively. The data showed that the level of phosphorylated mTOR, AKT and S6 K1 were significantly higher in HCC tumor tissues than those of adjacent normal liver tissues (Fig. 4a). And western blotting analysis showed that phosphorylated mTOR, AKT and S6 K1 level were dramatically higher in the HCC cell lines compared with those in the control group (Fig. 4b).What’s more, knockdown GOLPH3 down-regulated the phosphorylation of mTOR, AKT and S6 K1 in MHCC97L and MHCC97H (Fig. 5a). These results were further confirmed in xenograft tumor model (Fig. 5b). In our supplementary material, we also discovered that in HCC cell lines, knockdown GOLPH3 inhibited the expression of Raptor, which is a specific element of mTORC1. However, silencing GOLPH3 has no effect on the expression of Rictor, one component of mTORC2. (Additional file 1: Figure S1A). Collectively, these data provided strong biochemical evidence that GOLPH3 exerted a pivotal role in promoting the progression of HCC by activating mTOR signaling pathway.

## Discussion

This study demonstrated that GOLPH3 was significantly over-expressed in human HCC tumor tissues. In addition, high expression of GOLPH3 was closely correlated with the serum AFP level, a widely used serum marker of HCC. In vitro, we found that the down-regulation of GOLPH3 resulted in decreasing cell proliferation and increasing cell apoptosis. Moreover, diminished GOLPH3 could inhibit tumorigenic properties of HCC cells in vivo. Furthermore, mTOR signaling pathway was involved in the pathological mechanism of GOLPH3 in HCC.

Hepatocellular carcinoma (HCC) is a cancer with a high mortality rate due to the fact that diagnosis usually occurs at an advanced stage. So it remains a serious threat to human health. Over the past decades, no significant improvements have been achieved regarding the early diagnosis and treatment of HCC. The understanding of the molecular oncogenesis underlying HCC is still lacking. Many studies have showed that GOLPH3 is an oncogene, which is involved in tumorigenesis and correlated with poor prognosis. Furthermore, some previous studies report that GOLPH3 overexpression is associated with poor clinical outcome in HCC [25, 26]. In this study, we also discovered that GOLPH3 was high expressed in the HCC tumor tissues (Fig. 1). It is reported that HCC patients with AFP level ≤ 20 ng/ml may benefit the most from hepatectomy, but patients with AFP level > 20 ng/ml need comprehensive therapy, surgical resection and close follow-up examinations [27]. So we put the 20 ng/ml AFP as a standard. And the results showed that the up-regulation of GOLPH3 was strongly correlated with high AFP level (Table 1). What’s more, that low expression of GOLPH3 promoted HCC cell apoptosis and inhibited tumorigenesis was found by functional studies in vitro and in vivo (Fig. 2 and Fig. 3).

However, to our knowledge, the precise mechanisms underlying the relationship between GOLPH3 and HCC are still rarely understood. Only one research has indicated that GOLPH3 can play a critical role in HCC aggressiveness and angiogenesis by activating the NF-κB signaling pathway [26]. In 2009, Scott et al. demonstrated that GOLPH3 could promote tumor growth via activating mTOR signaling pathway [13]. But it is unclear that whether this phenomenon also occurs in HCC or not.

The mammalian target of rapamycin (mTOR), a phosphatidylinositol 3 kinase (PI3K)-related serine/theronine kinase, plays a central role in regulating cell growth, proliferation and survival, in part by regulating translation initiation. mTOR functions in two structural and functional distinct protein complexes: mTORC1, which also contains two positive regulatory subunits, Raptor and mLST8, and two negative regulators, PRAS40 and DEPTOR; and mTORC2, which also contains Rictor, mSin1 and Protor, and also mLST8 and DEPTOR [28–32]. As a part of mTOR complexes, mTORC1 directly activates S6 K1 (p70 ribosomal protein S6 kinase), phosphorylated at Thr389 [33, 34], the major rapamycin-sensitive site. S6 K1 clearly plays an important role in the regulation of cell growth, cell cycle progression and cell proliferation [31]. While mTORC2 was found to play a critical role in phosphorylating Akt (Ser473), which is one of the most important survival kinases, involved in regulating a wide array of cellular processes, including metabolism, growth, proliferation and apoptosis [35–37]. In the HCC tissue specimens, immunochemistry revealed that the protein expression of p-mTOR (Ser2448), p-Akt (Ser473), and p-S6 K1 (Thr389) in the HCC tumor tissues were significantly higher than those in the paired normal groups (Fig. 4a). These results were verified in HCC cell lines (Fig. 4b). What’s more, by knocking down the GOLPH3 expression in HCC cells, the expression of p-mTOR (Ser2448), p-Akt (Ser473) and p-S6 K1 (Thr389) were remarkably decreased in the HCC cell lines and in the xenograft tumor model (Fig. 5a and Fig. 5b). These results indicated that GOLPH3 could be involved in HCC tumorigenesis via activating both mTORC1 and mTORC2. For further study, we also noted that knockdown GOLPH3 in the HCC cell lines diminished expression of Raptor rather than Rictor (see Additional file 1: Figure S1A). Research has suggested that Raptor can regulate mTOR activity and functions as a scaffold for recruiting mTORC1 substrates [38]. Perhaps GOLPH3 could regulate Raptor to activate mTOR signaling cascade in HCC. Studies have demonstrated that mTOR controls NF-κB activity by stimulating IKK [39] and GOLPH3 could sustain the activation of the NF-κB signaling pathway in HCC [26]. Thus, the interplay among GOLPH3, mTOR and NF-κB in the progression of HCC is likely to be complex and needs to be further investigated. What’s more, research has shown that DNA-PK, a multimeric complex consisting of a catalytic subunit, DNA-PKcs, and the regulatory subunits, Ku70 and Ku86, plays a critical role in the DNA damage response, particularly in the recognition and repair of double stranded breaks [40]. DNA-PK could phosphorylate GOLPH3 and play an important role in DNA-PK/GOLPH3/MYO18A pathway to regulate cell survival following DNA damage [41]. In addition, Hemmings and colleagues observed that DNA-PK could phosphorylate AKT on Ser473 [42]. And in our study, we discovered that when knocking down the GOLPH3 expression in HCC cells, the expression of DNA-PKcs and Ku70 was decreased (Additional file 1: Figure S1B). Perhaps this was because GOLPH3 and DNA-PK could via regulating the phosphorylation of AKT influence each other in Golgi’s function during DNA damage response. The more exact mechanism between DNA-PK/GOLPH3/MYO18A pathway and GOLPH3/mTOR pathway to regulate function of GOLPH3 in HCC progression will be explored in our further studies. And we think it is likely that there exist one or more proteins involved in GOLPH3-associated xsignaling pathway in HCC which may have therapeutic utility.

## Conclusions

This study illustrates that the expression of GOPLH3 is pivotal in HCC by activating mTOR signaling pathway, which has emerged as an attractive therapeutic target for cancer therapy. Based on these results, we propose that the inhibition of GOLPH3-mTOR signaling pathway may represent a novel target for HCC therapy.

## Additional file

Additional file 1: **Figure S1.** GOLPH3 depletion inhibited Raptor and DNA-PK expression. (A) Western blotting of Raptor and Rictor in MHCC97L and MHCC97H cells transfected with si-Ctrl and si-GOLPH3. (B) Western blotting of DNA-PKcs, Ku70 and Ku86 in MHCC97L and MHCC97H cells transfected with si-Ctrl and si-GOLPH3. The expression levels were normalized to GAPDH. Data were presented as the mean ± SD. *P*-values were calculated using Student’s t-test, * *P* < 0.05, ** *P* < 0.01, *** *P* < 0.001. (TIF 9945 kb)

## Abbreviations

AFP: α-fetoprotein
DNA-PK: DNA damage protein kinase
GOLPH3: Golgi phosphoprotein 3
HCC: hepatocellular carcinoma
IHC: immunohistochemistry
mTOR: mammalian target of rapamycin
mTORC1: mammalian target of rapamycin complex 1
mTORC2: mammalian target of rapamycin complex 2
MTT: 3-(4,5-dimethylthiazol-2-yl)-2,5-diphenyltetrazolium bromide
qPCR: quantitative real-time PCR
Raptor: regulatory associated protein of mTOR
Rictor: rapamycin insensitive companion of mTOR
S6 K1: p70 ribosomal protein S6 kinase
