# Supplementary figures and images for: Golgi phosphoprotein 3 (GOLPH3) promotes hepatocellular carcinoma progression by activating mTOR signaling pathway
Source: BMC Cancer. 2018 Jun 18;18:661. doi: 10.1186/s12885-018-4458-7 (PMC6006993; doi:10.1186/s12885-018-4458-7)

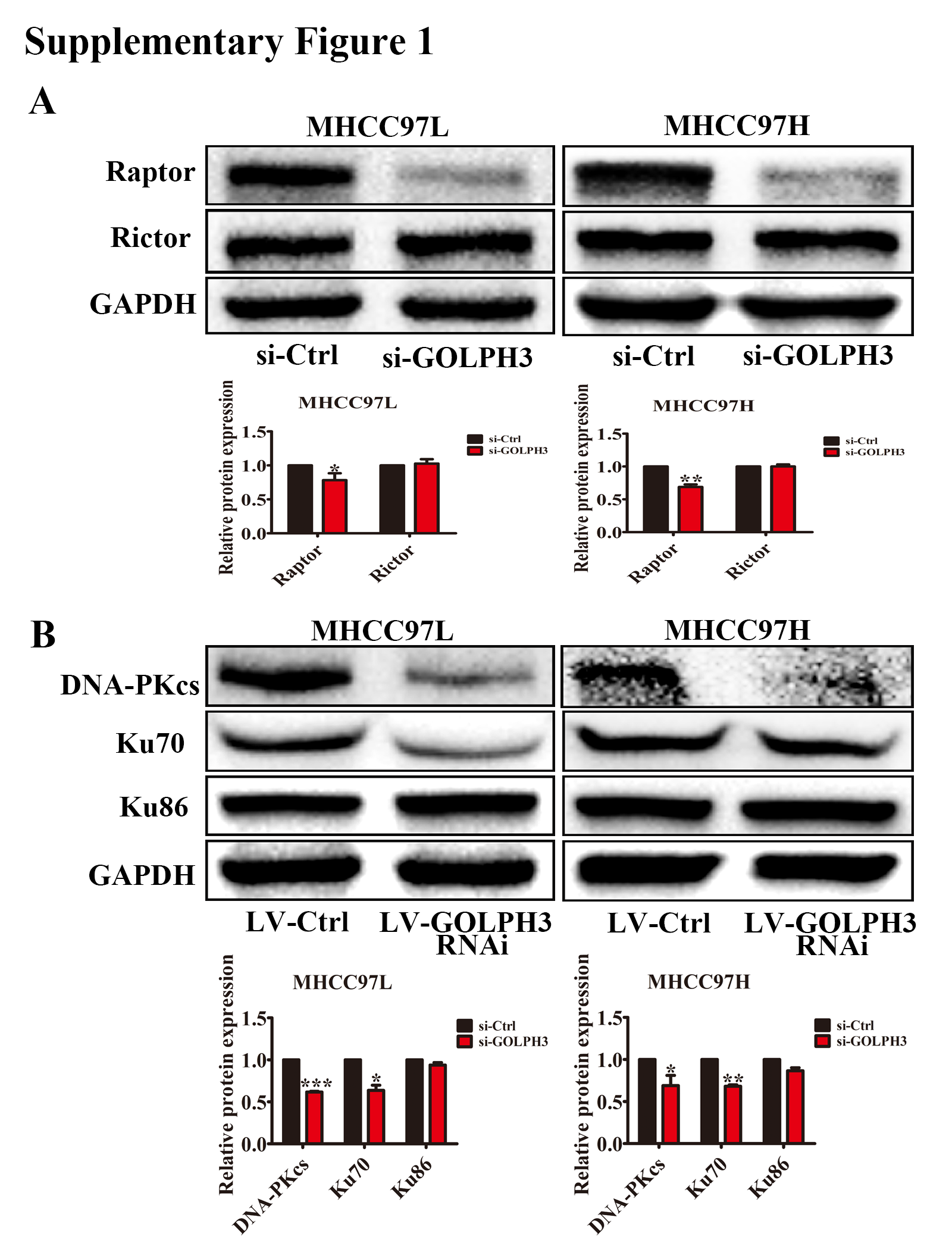

Supplement: Supplementary file 1 — Figure S1. GOLPH3 depletion inhibited Raptor and DNA-PK expression. (A) Western blotting of Raptor and Rictor in MHCC97L and MHCC97H cells transfected with si-Ctrl and si-GOLPH3. (B) Western blotting of DNA-PKcs, Ku70 and Ku86 in MHCC97L and MHCC97H cells transfected with si-Ctrl and si-GOLPH3. The expression levels were normalized to GAPDH. Data were presented as the mean ± SD. P-values were calculated using Student’s t-test, * P < 0.05, ** P < 0.01, *** P < 0.001. (TIF 9945 kb) [file 12885_2018_4458_MOESM1_ESM.tif]
